# Bidirectionally Oriented Carbon Fiber/Silicone Rubber Composites with a High Thermal Conductivity and Enhanced Electromagnetic Interference Shielding Effectiveness

**DOI:** 10.3390/ma16206736

**Published:** 2023-10-18

**Authors:** Jianan Song, Yicheng Fan, Anjun Shi

**Affiliations:** 1 School of Materials Science & Engineering, Jiangsu University, Zhenjiang 212000, China; songjianan@ujs.edu.cn; 2 Military Representative Office of the Armaments Department of the PLA Navy, Wuxi 214000, China; mjjfanyicheng@163.com

**Keywords:** orientation, carbon fiber, thermal conductivity, electromagnetic interference shielding

## Abstract

Effective thermal management and electromagnetic shielding have emerged as critical goals in contemporary electronic device development. However, effectively improving the thermal conductivity and electromagnetic shielding performance of polymer composites in multiple directions continues to pose significant challenges. In this work, inspired by the efficiency of interchange bridges in enabling vehicles to pass quickly in multiple directions, we employed a straightforward method to fabricate bidirectionally oriented carbon fiber (CF)/silicone rubber composites with an interchange-bridge-like structure. The high aspect ratio of CFs and their bidirectional orientation structure play a pivotal role in facilitating the formation of thermal and electrical pathways within the composites. Meanwhile, the bidirectionally oriented CF/silicone rubber composites showed a significant enhancement in tensile strength in both the vertical and horizontal directions, attributed to the cross-arrangement of CF arrays within the composites. At a filler content of 62.3 wt%, the bidirectionally oriented CF/silicone rubber composites had a high tensile strength of 6.18 MPa. The composites also exhibited an excellent thermal conductivity of 25.3 W/(m·K) and a remarkable electromagnetic interference shielding effectiveness of 61.6 dB. The bidirectionally oriented CF/silicone rubber composites show potential for addressing thermal management and electromagnetic shielding issues in electronic devices.

## 1. Introduction

With the rapid development of microelectronics technology, electromagnetic interference (EMI) and thermal accumulation have become important issues affecting the performance of microelectronic devices [1,2,3]. To address the issues of EMI and thermal accumulation, research on polymer-based composites with excellent thermal conductivity and electromagnetic shielding properties has emerged as a hot topic [4,5,6]. However, polymers inherently exhibit poor thermal conductivity and electromagnetic shielding capabilities, necessitating the incorporation of external fillers to enhance these properties [7,8,9]. Carbon-based fillers are commonly used as fillers for preparing thermally and electrically conductive polymer composites for thermal management and EMI shielding due to their high thermal conductivity and electrical conductivity properties [10,11,12]. For example, Yang et al. [13] improved the thermal conductivity and EMI shielding performance of polyethylene by filling it with expanded graphite. Chen et al. [14] successfully prepared a composite material consisting of reduced graphene oxide (rGO) and polymer, which exhibited a remarkable EMI shielding efficiency of 31.8 dB. Ata et al. [15] achieved exceptional EMI shielding effectiveness by preparing composites using carbon nanotubes (CNTs) and polycarbonate (PC) through injection molding. Their composites demonstrated the highest EMI shielding values ranging from 46.7 dB to 51.1 dB.

However, nanoscale carbon-based fillers tend to agglomerate, often leading to difficulties in dispersion and an increase in the viscosity of the polymer, making processing challenging [16]. Moreover, this can lead to a deterioration in the mechanical properties of the composites [17]. Recently, carbon fibers (CFs) have been utilized by researchers to fabricate thermally and electrically conductive composites with a high aspect ratio due to ease of processing, their ability to enhance the mechanical properties of composites, and their superior dispersibility compared to nanoscale carbon-based fillers [18,19,20]. Additionally, CFs can create extremely long thermal and electrical conduction pathways, which is advantageous for enhancing the thermal and EMI shielding performance of their polymer composites [21]. For example, Qi et al. [20] fabricated conductive Ag-plated CF/epoxy composites, which showed an EMI shielding effectiveness of 35–38 dB and exhibited a high thermal conductivity of 2.33 W/(m·K) at a filler content of 7.0 wt%. Wen et al. [22] improved the EMI shielding effectiveness of polyvinyl butyral film to 32 dB by incorporating Ni-graphite and short-cut CFs as fillers.

In order to improve the performance of polymer composites, researchers have employed various processing approaches to establish continuous pathways for thermal and electrical conduction within the polymer matrix [23,24]. Through the orientation of one-dimensional and two-dimensional carbon-based fillers, as well as the creation of a three-dimensional network structure, fillers interconnect with each other to form efficient pathways for thermal and electrical conduction [25]. Simultaneously, establishing continuous filler pathways helps to reduce the interfaces between the fillers and the polymer in thermal and electrical transport pathways [13,26]. For example, highly aligned CFs with continuous lengths were successfully prepared on a large scale through a modified papermaking method [27]. The highly aligned CFs increased the EMI shielding effectiveness of epoxy composites to 46.4 dB by optimizing mat layout configurations. Lee et al. [28] reported a composite material with a high thermal conductivity and strong electromagnetic wave shielding performance, which was engineered by vertically aligning carbon fibers (CFs) through the application of a perpendicular magnetic field. This unique manufacturing technique, optimized for efficient heat transfer in the vertical direction, led to an impressive thermal conductivity of 1.96 W/(m·K) and an EMI shielding effectiveness of 14.06 dB. Ren et al. [8] have conducted a pioneering study in which they showcased a meticulously engineered 3D carbonized loofah sponge with a conductive skeleton loaded with graphene nanosheets and CNTs. This unique structure was subsequently filled with epoxy-resin-modified cyanate ester composites. The resulting composite exhibited outstanding EMI shielding capabilities and commendable thermal conductivity.

However, despite the interconnected nature of the three-dimensional network structure, its degree of orientation is relatively low [29]. In oriented structural composites, both thermal conductivity and electromagnetic shielding performance exhibit a high degree of anisotropy [30]. Thermal conductivity performance is notably weaker in directions perpendicular to the orientation, while the electromagnetic shielding performance is also relatively poor along the orientation direction. Enhancing both the thermal conductivity and electromagnetic shielding performance of composites simultaneously in multiple directions remains a challenging task [31].

In this work, inspired by the efficiency of interchange bridges in enabling vehicles to pass quickly in multiple directions, a simple method was employed to fabricate interchange-bridge-like bidirectionally oriented CF/silicone rubber composites. CFs were used to provide continuous thermal and electrical conduction pathways. Silicone rubber was used as a polymer matrix due to its characteristics such as flexibility, corrosion resistance, chemical stability, and ease of processing [32]. By constructing a new bidirectionally oriented structure of CFs, simultaneous improvements in the thermal conductivity and EMI shielding performance of the silicone rubber composite material in multiple directions were achieved. This work can provide new ideas and methods for the structural design of thermally conductive and EMI shielding polymer composites.

## 2. Materials and Methods

### 2.1. Materials

Short-cut CFs with an average aspect ratio of 8.6 × 10^2^ were made by Toray Industries, Inc., Tokyo, Japan. Poly(ethylene glycol) monolaurate was provided by Shanghai Aladdin Bio-chem Technology Co. Ltd., Shanghai, China. Additive-type liquid silicone rubber was purchased from Jiangxi Bluestar Xinghuo Silicones Co., Ltd., Jiujiang, China. The platinum catalyst was made by Shanghai Guiyou New Material Technology Co., Ltd., Shanghai, China. 2-Methyl-3-butyn-2-ol was purchased from Sigma-Aldrich (Shanghai, China) Trading Co., Ltd., China.

### 2.2. Preparation of Bidirectionally Oriented CF/Silicone Rubber Composites

First, short-cut CF sheets were arranged in the mold along the same horizontal direction to form a layer, and then, above it, short-cut CF sheets were placed in the mold along the vertical direction to create another layer. These two steps were alternated until a certain thickness of the interchange CF array was achieved. Additive-type liquid silicone rubber was first mechanically mixed with the curing inhibitor 2-methyl-3-butyn-2-ol. Next, poly(ethylene glycol) monolaurate was added to the above mixture as an interface modifier. Subsequently, the platinum catalyst was further blended into the mixture. A silicone rubber compound was formed by uniformly mixing additive-type liquid silicone rubber, poly(ethylene glycol) monolaurate, platinum catalyst, and 2-methyl-3-butyn-2-ol in a ratio of 100:1:0.1:0.1 through mechanical agitation. The silicone rubber compound was poured into the mold containing the CF array, where it was initially immersed at room temperature for 1 h and then transferred to a vacuum oven and immersed for 10 h to ensure that the voids within the CF array were filled with silicone rubber. Finally, different pressures were applied to extrude varying quantities of silicone rubber, which was then heated at 120 °C for 20 min to cure, resulting in the bidirectionally oriented CF/silicone rubber composites. The obtained composites can be cut and flipped to yield CF/silicone rubber composites with dual orientations, both vertical and horizontal. For the purpose of comparison, CF/silicone rubber composites with a single directional orientation were also prepared.

### 2.3. Characterization

An FEI Nova Nano450 field emission scanning electron microscope (SEM) was utilized to observe the morphology and microstructure of the bidirectionally oriented CF/silicone rubber composites. Mechanical properties were assessed using an Instron 4465 machine (Instron, Boston, MA, USA), with tensile properties evaluated following ASTM D412 standards, and tear strength determined in accordance with ASTM D624 standards. Tensile tests employed dumbbell-shaped specimens, while right-angle specimens were used for tear strength testing. Thermogravimetric analysis (TGA) curves were obtained using a thermogravimetric analyzer (TA, Q5000IR, New Castle, DE, USA) under a nitrogen atmosphere, with a heating rate of 20 °C per minute, spanning from 50 °C to 700 °C. Thermal conductivities of the composites were measured using a Netzsch LFA467 laser flash analyzer (Netzsch, Selb, Germany), employing the laser flash method. Electrical conductivity was assessed using an FT-340 double electric four-probe resistance ratio tester (Rooko, Ningbo, China). To measure the electromagnetic interference (EMI) and the shielding effectiveness of the bidirectionally oriented CF/silicone rubber composites, a vector network analyzer (Anritsu MS4644A, Atsugi, Japan) was employed in the frequency range of 8.2–12.4 GHz, utilizing the waveguide method.

## 3. Results and Discussion

### 3.1. Preparation of Bidirectionally Oriented CF/Silicone Rubber Composites

**Figure 1:**
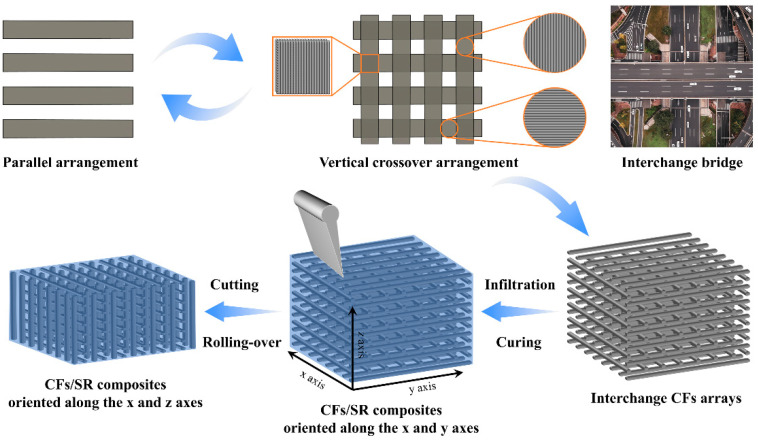
Schematic diagram of the preparation of bidirectionally oriented CF/silicone rubber composites.

Inspired by the effectiveness of interchange bridges in facilitating multidirectional traffic flow for automobiles, this work aims to construct a bidirectional filler structure within a polymer matrix. This was executed to simultaneously enhance the vertical and horizontal thermal conductivity and EMI shielding properties. The preparation process of the composite material is illustrated in Figure 1. Initially, CF sheets were arranged horizontally, followed by the vertical cross-arrangement of CF sheets on the horizontally aligned CF layer. This sequence was alternately repeated to create an interchange CF array. Subsequently, the CF array was impregnated with silicone rubber and then cured under different pressures. Finally, the resulting composite material was cut and rotated to obtain a bidirectionally oriented CF/silicone rubber composite. Poly(ethylene glycol) monolaurate was added to the silicone rubber compound to enhance the interface interaction between the CFs and silicone rubber. By applying varying pressures during the curing process, CF/silicone rubber composites with different levels of CF compaction were obtained, resulting in CF/silicone rubber composites with varying filler contents.

### 3.2. Microstructure of Bidirectionally Oriented CF/Silicone Rubber Composites

**Figure 2:**
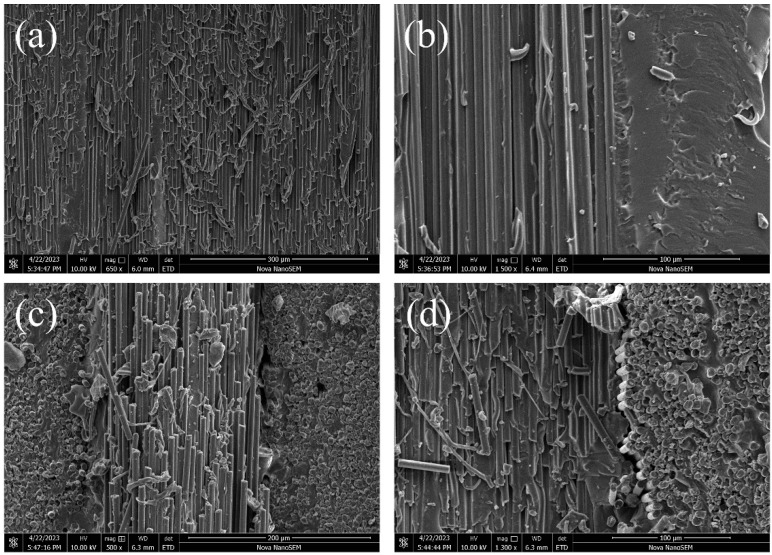
SEM images of oriented CF/silicone rubber composites: (**a**,**b**) unidirectional orientation, (**c**,**d**) bidirectional orientation.

The SEM images in Figure 2 illustrate the morphologies of the unidirectionally and bidirectionally oriented CF/silicone rubber composites. It can be observed from Figure 2a that CFs have an extremely high aspect ratio and form a precisely oriented structure within the composites. This high aspect ratio and orientation play a pivotal role in facilitating the formation of thermal and electrical pathways within the composites. Additionally, due to curing under pressure, CF layers were tightly stacked, creating more pathway structures within the composites. As seen in Figure 2b, the addition of poly(ethylene glycol) monolaurate enhanced the interface interaction between CFs and silicone rubber, resulting in a tight bond at the interface with no apparent defects. As shown in Figure 2c,d, in the case of bidirectionally oriented composites, CF layers were arranged in a cross pattern, both from the bottom to the top and from the inside out. These bidirectional thermal and electrical pathways could simultaneously improve the heat conduction performance and electromagnetic shielding properties in both directions.

### 3.3. Mechanical Properties of Bidirectionally Oriented CF/Silicone Rubber Composites

**Figure 3:**
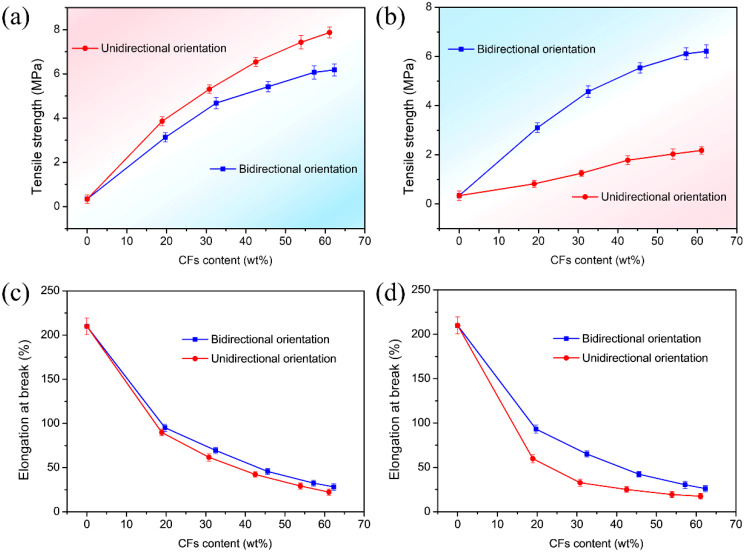
(**a**,**c**) Mechanical properties of the unidirectionally oriented CF/silicone rubber composites along the CF orientation and the bidirectionally oriented CF/silicone rubber composites along the horizontal orientation of CFs: (**a**) tensile strength, (**c**) elongation at break. (**b**,**d**) Mechanical properties of the unidirectionally oriented CF/silicone rubber composites perpendicular to the CFs orientation and the bidirectionally oriented CF/silicone rubber composites along the vertical orientation of CFs: (**b**) tensile strength, (**d**) elongation at break.

Figure 3 shows the mechanical properties of the unidirectionally and bidirectionally oriented CF/silicone rubber composites. From Figure 3a, it can be observed that the tensile strength in the CF orientation direction of both types of composites increase with an increase in filler content. The unidirectionally oriented CF/silicone rubber composites exhibited higher tensile strengths along the CF orientation direction compared to the bidirectionally oriented CF/silicone rubber composites along the horizontal orientation of CFs. For the unidirectionally oriented composites, at a filler content of 61.1 wt%, the tensile strength along the CF orientation direction was 7.87 MPa. The tensile strength of the bidirectionally oriented composites in the horizontal orientation increased rapidly at lower filler contents but exhibited a slower rate of increase at higher filler contents. For the bidirectionally oriented composites, at a filler content of 62.3 wt%, the tensile strength in the horizontal orientation was 6.18 MPa. The mechanical properties of the unidirectionally oriented composites in the direction perpendicular to the orientation are also performance aspects that need attention. The filler content of 62.3 wt% yielded the most optimal mechanical performance due to its maximization of the reinforcement effect provided by the CFs within the composite. The composite featured the greatest density of CFs, and when these fibers were correctly oriented along the tensile direction, they significantly enhanced the composite’s resistance to mechanical stress and strain. The aligned carbon fibers along the tensile direction played a crucial role in enhancing the mechanical performance of the composite. Figure 3b reveals that unidirectionally oriented composites exhibit a noticeably lower tensile strength in the direction perpendicular to their orientation compared to their tensile strength along the orientation direction. At a filler content of 61.1 wt%, the tensile strength in the perpendicular direction was only 2.18 MPa. In contrast, bidirectionally oriented composites demonstrated a nearly identical tensile strength in both the vertical and horizontal orientations. The bidirectionally oriented CF/silicone rubber composites showed a significant enhancement in tensile strength in both directions, attributed to the cross arrangement of CF arrays within the composites.

Figure 3c illustrates the variation in elongation at break along the orientation direction for unidirectionally oriented CF/silicone rubber composites as a function of filler content, as well as the elongation at break in the horizontal orientation for bidirectionally oriented CF/silicone rubber composites with changing filler content. It could be clearly observed that the elongation at break of both composites sharply decreased as the filler content increased, which could be attributed to the rigidity of carbon fibers, making it difficult for the composites to undergo plastic deformation. For unidirectionally oriented CF/silicone rubber composites, at a filler content of 61.1%, the elongation at break in the orientation direction was 22.3%. On the other hand, bidirectionally oriented CF/silicone rubber composites, at a filler content of 62.3%, exhibited an elongation at break of 26.1% in the horizontal orientation. Figure 3d displays the change in elongation at break perpendicular to the orientation direction for unidirectionally oriented CF/silicone rubber composites and the elongation at break of bidirectionally oriented CF/silicone rubber composites in the vertical orientation with varying filler contents. Notably, bidirectionally oriented CF/silicone rubber composites exhibited nearly identical elongation at break in both the vertical and horizontal orientations. Unidirectionally oriented CF/silicone rubber composites showed a lower elongation at break in the direction perpendicular to the orientation compared to the orientation direction itself, mainly due to a significant decrease in tensile strength in the perpendicular direction, making the composites prone to fracture. The mechanical performance results of the composites indicated a significant improvement in mechanical properties in multiple directions for bidirectionally oriented CF/silicone rubber composites when compared to unidirectionally oriented CF/silicone rubber composites.

### 3.4. Thermal Properties of Bidirectionally Oriented CF/Silicone Rubber Composites

**Figure 4:**
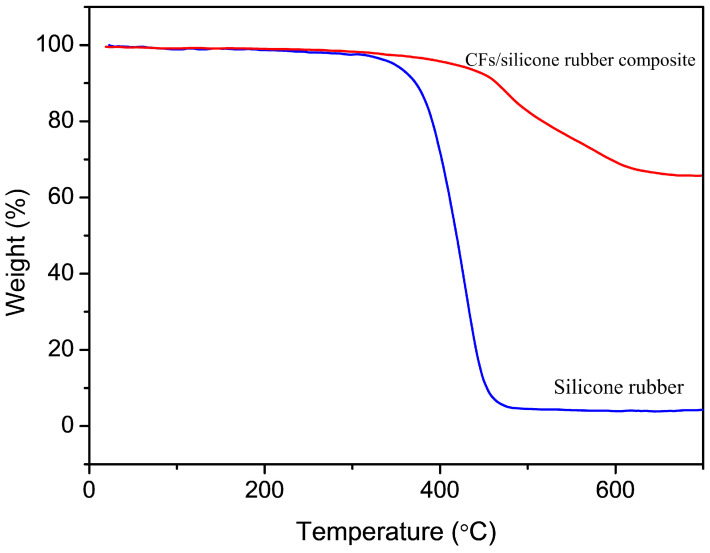
TGA curves of silicone rubber and the bidirectionally oriented CF/silicone rubber composite.

As shown in Figure 4, the temperature at 10% weight loss of silicone rubber is 372.3 °C, whereas the temperature at 10% weight loss of the bidirectionally oriented CF/silicone rubber composite is 465.5 °C. According to the TGA results, it is evident that the thermal decomposition temperature of the bidirectionally oriented CF/silicone rubber composite was increased, indicating a significant enhancement in thermal stability.

**Figure 5:**
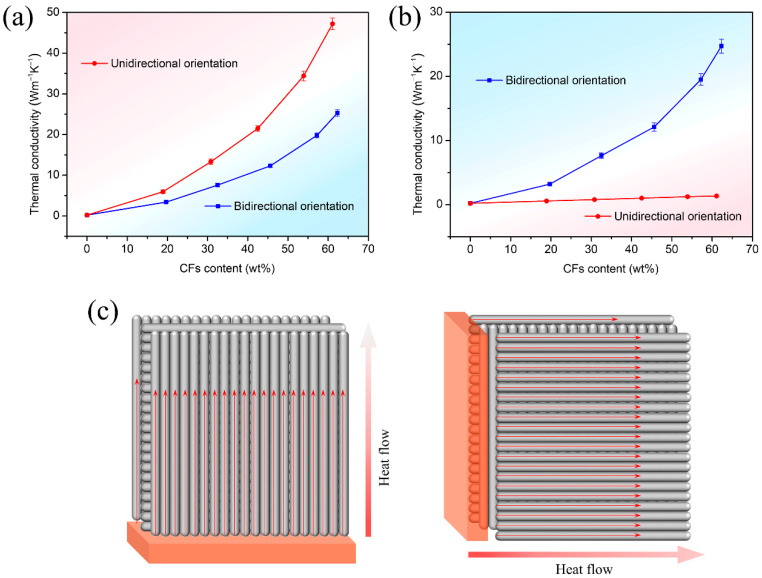
(**a**) Thermal conductivities of the unidirectionally oriented CF/silicone rubber composites along the CFs orientation and the bidirectionally oriented CF/silicone rubber composites along the horizontal orientation of CFs, (**b**) Thermal conductivities of the unidirectionally oriented CF/silicone rubber composites perpendicular to the CFs orientation and the bidirectionally oriented CF/silicone rubber composites along the vertical orientation of CFs, (**c**) Thermal conduction mechanisms of the bidirectionally oriented CF/silicone rubber composites.

Figure 5a depicts the variation in thermal conductivity along the orientation direction for unidirectionally oriented CF/silicone rubber composites as a function of filler content and the change in thermal conductivity along the horizontal orientation for bidirectionally oriented CF/silicone rubber composites with varying filler content. It can be observed that the thermal conductivity of both types of composites significantly increased with the increase in CF content. This effect can be attributed to the highly organized alignment of CFs, which provides efficient heat transfer pathways within the composites. Unidirectionally oriented CF/silicone rubber composites exhibited a higher thermal conductivity along the orientation direction compared to bidirectionally oriented CF/silicone rubber composites in the horizontal orientation. This difference arises because unidirectionally oriented CF/silicone rubber composites with the same filler content establish more effective heat transfer pathways along the orientation direction. Specifically, for unidirectionally oriented CF/silicone rubber composites, at a filler content of 61.1 wt%, the thermal conductivity along the orientation direction was 47.2 W/(m·K), while bidirectionally oriented CF/silicone rubber composites, at a filler content of 62.3 wt%, exhibited a thermal conductivity of 25.3 W/(m·K) in the horizontal orientation.

Figure 5b illustrates the change in thermal conductivity perpendicular to the orientation direction for unidirectionally oriented CF/silicone rubber composites and the variation in thermal conductivity in the vertical orientation for bidirectionally oriented CF/silicone rubber composites as the filler content changes. Remarkably, bidirectionally oriented CF/silicone rubber composites exhibited nearly identical thermal conductivity in both the vertical and horizontal orientations. Conversely, unidirectionally oriented CF/silicone rubber composites exhibited a significantly lower thermal conductivity in the direction perpendicular to the orientation compared to the orientation direction. Specifically, for unidirectionally oriented CF/silicone rubber composites, at a filler content of 61.1 wt%, the thermal conductivity in the direction perpendicular to the orientation was only 1.34 W/(m·K). This discrepancy arises because heat transfer within the composite material in the direction perpendicular to the orientation occurs along the diameter of the CFs, which inherently possess low thermal conductivity in the diameter direction. Furthermore, the thermal resistance at the interface between CF and silicone rubber further diminishes the composite’s thermal conductivity. The thermal conductivity results of the composites underscore that bidirectionally oriented CF/silicone rubber composites outperform unidirectionally oriented CF/silicone rubber composites by simultaneously enhancing the thermal conductivity in both the horizontal and vertical directions. 

Figure 5c provides a schematic representation of the thermal conduction mechanisms of the bidirectionally oriented CF/silicone rubber composites in both the horizontal and vertical directions. The bidirectionally oriented CF/silicone rubber composites exhibited a highly oriented arrangement of CFs in both horizontal and vertical directions. This unique structure could provide continuous and efficient heat conduction pathways in both orientations, leading to an improvement in thermal conductivity in both the horizontal and vertical directions. Furthermore, the high aspect ratio of CF reduces the number of interfaces through which heat Cn be transferred, resulting in a reduction in interfacial thermal resistance and further enhancing the overall thermal conductivity of the composites.

**Figure 6:**
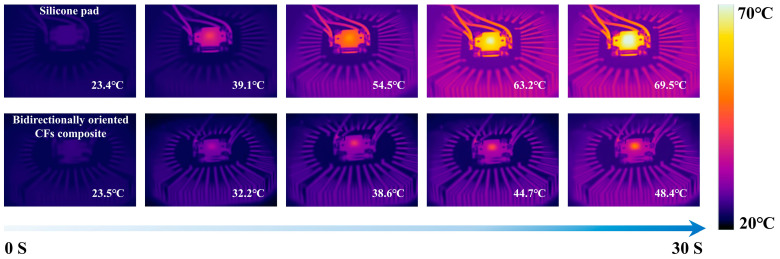
Infrared images of LEDs using a silicone pad and the bidirectionally oriented CF/silicone rubber composite as thermal interface materials.

To illustrate an application in thermal management, the bidirectionally oriented CF/silicone rubber composite and a commercial silicone pad (with a thermal conductivity of 5.0 W∙m^−1^∙K^−1^) were used as thermal interface materials (TIMs) between an LED chip and a heat sink, as shown in Figure 6. The results revealed that, after 30 s of operation, the LED’s surface temperature when the bidirectionally oriented CF/silicone rubber composite was employed as the TIM was 48.4 °C, whereas the surface temperature of the LED when the silicone pad was used as the TIM was 69.5 °C. These findings clearly demonstrate the enhanced thermal management capabilities of the bidirectionally oriented CF/silicone rubber composite.

### 3.5. EMI Shielding Performance of Bidirectionally Oriented CF/Silicone Rubber Composites

**Figure 7:**
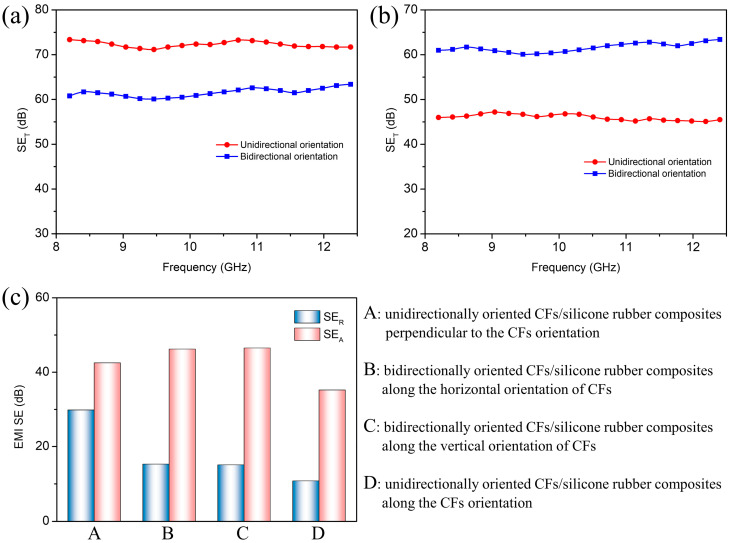
(**a**) SE_T_ of the unidirectionally oriented CF/silicone rubber composites perpendicular to the CF orientation and the bidirectionally oriented CF/silicone rubber composites along the horizontal orientation of CFs; (**b**) SE_T_ of the unidirectionally oriented CF/silicone rubber composites along the CF orientation and the bidirectionally oriented CF/silicone rubber composites along the vertical orientation of CFs; (**c**) SE_R_ and SE_A_ of the unidirectionally oriented and the bidirectionally oriented CF/silicone rubber composites.

Figure 7a shows the total EMI shielding effectiveness (SE_T_) values of the unidirectionally oriented CF/silicone rubber composites with 61.1 wt% filler perpendicular to the CF orientation and the bidirectionally oriented CF/silicone rubber composites with 62.3 wt% along the horizontal orientation of CFs over the X-band frequency ranges (8.2–12.4 GHz). Both composites exhibited remarkably high EMI SE_T_ values, with unidirectionally oriented CF/silicone rubber composites achieving an SE_T_ of 72.3 dB in the direction perpendicular to the orientation and the bidirectionally oriented CF/silicone rubber composites attaining 61.6 dB in the horizontal orientation. The oriented CF/silicone rubber composite structure led to an exceptional electrical conductivity, enabling the absorption of electromagnetic waves via electric loss. Figure 7b presents the EMI shielding performance of the unidirectionally oriented CF/silicone rubber composites with 61.1 wt% filler in the orientation direction and the bidirectionally oriented CF/silicone rubber composites with 62.3 wt% in the vertical orientation direction. The bidirectionally oriented CF/silicone rubber composites had nearly identical EMI SE_T_ values in both the vertical and horizontal orientations. In contrast, the unidirectionally oriented CF/silicone rubber composites displayed a lower EMI SE_T_ in the orientation direction compared to the direction perpendicular to the orientation. At a filler content of 61.1%, the unidirectionally oriented CF/silicone rubber composites exhibited an EMI SE_T_ of 46.0 dB in the orientation direction. This difference is a result of the reduced electrical conductivity at the material’s surface when incident waves approach along the direction of carbon fiber orientation, thereby inhibiting the occurrence of reflection loss. Figure 7c provides the values for the reflection loss (SE_R_) and absorption loss (SE_A_) of the composites, demonstrating that the bidirectionally oriented CF/silicone rubber composites have a reduced SE_R_ while maintaining a high EMI SE_T_.

**Figure 8:**
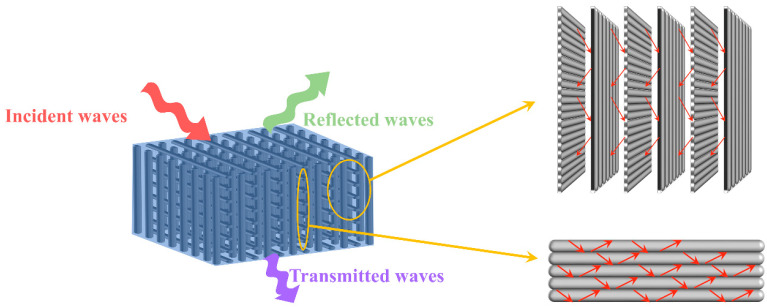
EMI shielding mechanism of the bidirectionally oriented CF/silicone rubber composites.

Figure 8 presents a schematic representation of the EMI shielding mechanism in the bidirectionally oriented CF/silicone rubber composites. The diagram reveals that, apart from conduction loss, electromagnetic waves within the composites may undergo multiple reflections and scattering within the interchange CF array, resulting in partial loss. The CF array can enhance the electromagnetic wave attenuation capacity of the composites through an absorption–reflection–absorption process.

Table 1 provides a comprehensive comparison of the thermal conductivities and EMI SE values among various polymeric composites incorporating CF-based fillers [22,27,32,33,34,35,36]. The data clearly illustrate that the bidirectionally oriented CF/silicone rubber composite outperforms other composites documented in the literature regarding the increases in thermal conductivity and EMI SE. These findings underscore the effectiveness of establishing a bidirectionally orientated structure for improving both the thermal conductivity and EMI shielding effectiveness.

## 4. Conclusions

In summary, inspired by the effectiveness of interchange bridges in facilitating multidirectional traffic flow for automobiles, interchange-bridge-like bidirectionally oriented CF/silicone rubber composites were prepared. The presented work investigated the microstructure and mechanical, thermal, and EMI shielding properties of the bidirectionally oriented CF/silicone rubber composites. The bidirectionally oriented CF/silicone rubber composites demonstrated nearly equivalent tensile strengths, thermal conductivities, and EMI shielding properties in both the horizontal and vertical directions. In contrast, unidirectionally oriented CF/silicone rubber composites exhibited significant anisotropy in these properties. At a filler content of 62.3 wt%, the bidirectionally oriented CF/silicone rubber composites showcased impressive properties, including a robust tensile strength of 6.18 MPa, an excellent thermal conductivity of 25.3 W/(m·K), and a remarkable EMI shielding effectiveness of 61.6 dB. The enhancement in these properties was attributed to the interchange structure of CFs within the silicone rubber. This study can provide insights and a foundation for the structural design and investigation of thermally conductive and EMI shielding composites.

## Figures and Tables

**Table 1:** Comparison of the thermal conductivities and EMI SE values of polymeric composites with CF-based fillers [22,27,32,33,34,35,36].

Filler	Matrix	Filler Content	TC (W∙m^−1^∙K^−1^)	SE (dB)	Ref.
Ni-graphite/CFs	Polyvinyl butyral	45 wt%	/	32.0	[22]
CFs	epoxy	40 vol%	/	46.4	[27]
CF/Mxene	epoxy	25 wt%	1.96	14.0	[28]
CF/Al_2_O_3_	Silicone rubber	40.5 vol%	8.44	40.8	[32]
CNTs/CFs	epoxy	34.5 wt%	7.50	38.4	[33]
NiCO@CFs	Silicone rubber	53 wt%	15.5	55.2	[34]
CFs	Silicone rubber	9.0 vol%	4.72	/	[35]
Ni-CFs	Polyether ether ketone	40 wt%	0.59	29.0	[36]
CFs	Silicone rubber	62.3 wt%	25.3	61.6	This work

## Data Availability

Not applicable.
